# DNA Hybridization Sensors Based on Electrochemical Impedance Spectroscopy as a Detection Tool

**DOI:** 10.3390/s91209513

**Published:** 2009-11-26

**Authors:** Jin-Young Park, Su-Moon Park

**Affiliations:** 1 Department of Chemistry, Pohang University of Science & Technology, Pohang 790-784, Korea; E-Mail: parkjin@postech.edu; 2 Interdisciplinary School of Green Energy Engineering, Ulsan National Institute of Science & Technology, Ulsan 689-805, Korea

**Keywords:** DNA, self-assembled monolayers, conducting polymers, nanostructures, EIS

## Abstract

Recent advances in label free DNA hybridization sensors employing electrochemical impedance spectroscopy (EIS) as a detection tool are reviewed. These sensors are based on the modulation of the blocking ability of an electrode modified with a probe DNA by an analyte, *i.e.*, target DNA. The probe DNA is immobilized on a self-assembled monolayer, a conducting polymer film, or a layer of nanostructures on the electrode such that desired probe DNA would selectively hybridize with target DNA. The rate of charge transfer from the electrode thus modified to a redox indicator, e.g., [Fe(CN)_6_]^3−/4−^, which is measured by EIS in the form of charge transfer resistance (*R*_ct_), is modulated by whether or not, as well as how much, the intended target DNA is selectively hybridized. Efforts made to enhance the selectivity as well as the sensitivity of DNA sensors and to reduce the EIS measurement time are briefly described along with brief future perspectives in developing DNA sensors.

## 1. Introduction

A DNA sensor based on electrochemical impedance spectroscopy (EIS) detection is a device that transduces changes in interfacial properties between the electrode and the electrolyte induced by DNA hybridization, conformational changes, or DNA damages to an electrical signal. In most DNA sensors, the target DNA must be labeled with a fluorophore [1-3], magnetic beads [4], or an enzyme [5] for its detection. On the contrary, DNA sensors based on EIS detection are label-free and, thus, possess advantages of low cost, simplicity, and ease of miniaturization. As a result, the interfacial impedance, which is obtained upon application of a small AC voltage overlaid on a DC bias potential to the sensing electrode and the AC current obtained thereof in the steady state, has been frequently employed for sensing various analytes by biosensors. Biosensors of these types have been constructed for the immunoassay and enzyme-based biosensing, as well as DNA and microorganism analyses; the subject has recently been reviewed by Guan *et al.* [6]. Other electrochemical techniques used for detection include cyclic voltammetry [7,8], differential pulse voltammetry [9-13], and amperometry [14], which have also been investigated for improvement of their sensitivity. However, when the differences in current are not significant in a low target concentration range, the EIS technique is more favorable than other electrochemical detection techniques in that significantly large differences are usually obtained due to the inverse relation of the impedance with the current, *i.e.*, Δ*R*(*Z*) = *ΔV*/*ΔI*.

**Figure 1.**
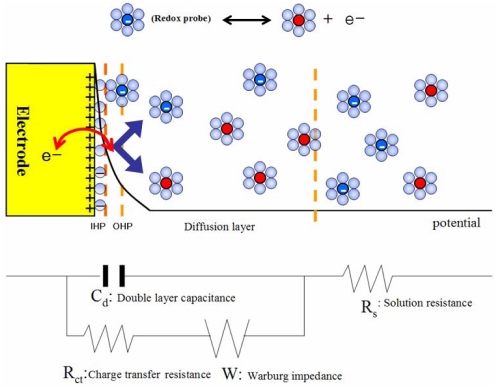
Schematic diagram for an electrode/electrolyte interface in a faradaic sensor and its exemplary model circuit [15].

An electrode/electrolyte interface may be simplified as shown by the schematic diagram in Figure 1. When an electrode is electrified by an applied DC potential, solvated counter ions form an electrical double layer by aligning along the electrode surface, which is represented by the so-called double layer capacitor with a capacitance of *C*_d_. The electron transfer to/from the electroactive species, which may approach the electrode as close as two solvent molecules away in the outer Helmholtz plane (OHP), takes place across the inner Helmholtz plane (IHP) by overcoming the activation barrier, *R*_p_, which is termed a polarization resistance, and the solution resistance, *R*_s_. Once the electron transfer gets started, the Warburg impedance (*W*) due to the mass transport begins to play a role in determining the electrode kinetics. In the non-faradaic EIS detection with no redox indicator added, the capacitance or the dielectric constant of the probe layer can be utilized as a main sensing signal. The charge-transfer resistance (*R*_ct_), which is the polarization resistance at an equilibrium potential, is utilized as a main indicator in the faradaic EIS detection. A redox pair such as [Fe(CN)_6_]^3-/4-^ or [Ru(NH_3_)_6_]^2+/3+^ is frequently used as a redox indicator for the electrode kinetics at the interface, which is modified by a substrate layer as well as probe and target DNAs on the electrode surface. Thus, the *R*_ct_ values indicate how crowded the electrode surface is when it is modified by a functional molecule, which is capable of selectively capturing a given analyte. The *R*_ct_ values are determined by how selective binding has taken place with the analyte and how much analyte is in the test solution.

The changes in resistances or capacitances of the interface are induced by the DNA hybridization events with a single-stranded target DNA on a suitably designed probe platform. To improve the performance of the DNA sensor, the probe layer should be constructed using a well-defined surface chemistry preventing the non-specific binding as well as other side reactions so that it would exhibit high selectivity for a specific target DNA. As a result, various DNA sensors have been embodied on the electrodes modified with the various platforms such as self-assembled monolayers (SAMs), mixed SAMs, conducting polymer films, various nanomaterials such as gold nanoparticles, and peptide nucleic acids. The design of the probe layer depends on whether the sensor is of faradaic or non-faradaic nature. For example, a compact SAM or an insulating layer without a leakage current is needed for non-faradaic signal processing while a less-packed SAM or a conductive layer accessible to the redox species are more desirable for a faradaic sensor. Changes in electrical properties occurring at the DNA probe layer are usually extracted using a best fitting model. Each circuit element obtained by fitting impedance responses to an electrical circuit can be utilized for analyzing the type and the amount of target DNA as well as its conformational changes [16,17]. In general, the changes in impedances are linearly related to the surface coverage by a target analyte in a low concentration region, but sometimes it is logarithmically related to the amount for the target analyte in the case of high concentration ranges or the heterogeneous binding between the probe and the target molecules. In a faradaic sensor, the charge-transfer resistance, *R*_ct_, is associated with the energy barrier for electron transfer to/from the redox indicator approaching to the electrode surface, which is determined by the change in the crowdedness of the probe layer caused by its binding with target DNA. In a non-faradaic sensor, the capacitance of the probe layer is a main indicator exhibiting the conformational changes of double-stranded DNA due to its hybridization. Katz and Willner have published an excellent review on various biosensors including immunosensors, enzyme based sensors, and DNA sensors employing faradaic and non-faradaic impedance spectroscopy as a detection tool [18]. Berggren *et al.* also introduced the non-faradaic capacitive biosensors [19].

In the present review, we focus on recently developed DNA sensors constructed on various classes of materials used for modifying electrode surfaces such as self-assembled monolayers, conducting polymers, and nanomaterials, which show high sensitivity and selectivity for various target DNAs using faradaic and non-faradaic impedance spectroscopy as a detection tool. We limit our discussions to DNA as a target analyte and EIS as a detection tool.

## 2. DNA Sensors Based on Self-Assembled Monolayers

Monitoring *R*_ct_ values on SAM modified gold electrode surfaces supporting thiolated probe DNAs is perhaps the most convenient and important method for investigating the DNA hybridization and conformational changes. Thiolated probe DNAs form a self-assembled monolayer on the gold surface due to the high affinity of the thiol group towards gold. Ito *et al.* detected changes in *R*_ct_ values according to the location of the mismatched pair in the DNA duplex on the thiolated probe DNA SAM [20]. To analyze the DNA hybridization kinetics, Li *et al.* attempted to make real-time measurements of *R*_ct_ values and reported an affinity constant of 10^8^ M^−1^ for a perfect match [21]. On the immobilization of a probe DNA molecule with a disulfide, the Li and Kraatz group characterized the B-DNA (native DNA) and M-DNA formed by adding Zn^2+^ to B-DNA [22-26]. The different charge and conformational characteristics of B-DNA and M-DNA on the surface led to different charge-transfer resistances for the redox indicator ions. Thus, they discriminated target DNA with one mismatch against a complementary DNA based on the difference in the *R*_ct_ values between M-DNA and B-DNA in presence of the [Fe(CN)_6_]^3−/4−^ redox indicator. Later, they examined eight metal ions including alkaline earth metal ions (Mg^2+^ and Ca^2+^), trivalent ions (Al^3+^ and La^3+^), and divalent transition metal ions (Ni^2+^, Cu^2+^, Cd^2+^, and Hg^2+^) and reported that these metal ions cause decreases in *R*_ct_ values. The difference of the charge transfer resistance (Δ*R*_ct_) of ds-DNA films in the presence and absence of these metal ions was different and particular to a given metal ion [27]. Further, the Δ*R*_ct_ values for ds-DNA films with a single A-C mismatch are smaller than those of fully matched ds-DNA films for Ca^2+^.

The charge-transfer resistance to the redox indicator is governed by surface blocking effects exerted by the hybridization of target DNA with the probe and the charged state of the probe layer on the electrode. In efforts to amplify the sensing signal, *i.e.*, the *R*_ct_ value, target DNA tagged with negatively charged liposome was utilized to enhance the repulsion between the target DNA and the negative charged redox indicator ions, *i.e.*, [Fe(CN)_6_]^3-/4-^, leading to detection limits of 1 × 10^−12^ ∼ 1 × 10^−13^ M [28,29]. To enhance the blocking ability of the sensing electrode for more sensitive detection of hybridization, an insulating layer formed by precipitation of an enzyme-catalyzed insoluble product was taken advantage of [28,29]. In this case, the pathway of electron transfer to/from the electrode was very effectively blocked and changes in *R*_ct_ values were remarkably enhanced upon hybridization leading to a low detection limit of 1.2 pM.

**Figure 2.**
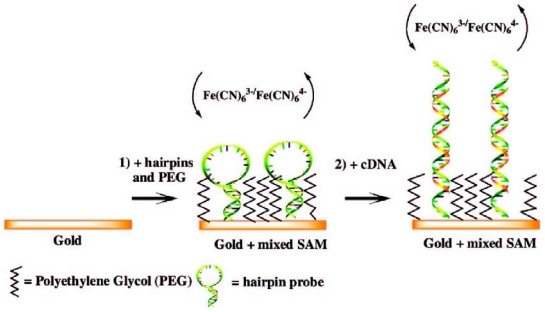
(1) The immobilization of a hairpin structured probe and PEG onto the gold surface and (2) hybridization with a complementary target [31].

Immobilization of thiolated probe DNA on the gold surface with an optimum density is a key technique for improving DNA sensing efficiencies. To control the radii around the probe DNAs to secure appropriate space for hybridization, mixed SAMs obtained from an appropriate ratio of thiolated probe DNA molecules and other blocking thiols have been frequently utilized. Since Herne and Tarlov attempted to prepare a mixed SAM of thiolated ss-DNA and mercaptohexanol (MCH) for more effective hybridization in their early study [30], two-step methods, in which probe DNA is first immobilized on the surface followed by the formation of the MCH SAM, have been widely employed for DNA sensors [16,21,31]. Keighley *et al.* [32] studied the possibility of obtaining an optimum density of immobilized probe DNAs on the gold surface by simultaneously immobilizing both thiolated probe DNA and MCH instead of using the two-step method introduced by Herne and Tarlov. Based on EIS measurements, they found that the degree of increase in the charge-transfer resistance for the [Fe(CN)_6_]^3−/4−^ redox indicator during the hybridization is related to the probe density obtained by controlling the fraction of thiolated probe DNA in the mixed solution. As a result, the increase in charge-transfer resistance was observed only above a threshold probe surface density of 2.5 × 10^12^ cm^−2^ due to the repulsion between negatively charged DNA and the negative redox indicator ions, *i.e.*, the [Fe(CN)_6_]^3−/4−^ pair. The optimum probe density was found to be 5.4 × 10^12^ cm^−2^, which was obtained from a solution containing both thoiolated DNA and MCH with a DNA mole fraction of 20%. Kjällman *et al.* employed thiolated polyethylene glycol [PEG: CH_3_-(CH_2_CH_2_O)_6_-CH_2_CH_2_SH] as an alternative for MCH for controlling the probe density and blocking the non-specific binding [33]. On the mixed SAM consisting of PEG and the hairpin DNA probe as displayed in Figure 2, the conformational change of the hairpin probe caused by hybridization induced an increase or decrease in the *R*_ct_ value for electron transfer to/from Fe(CN_6_)^3−/4−^. This strategy lowered the detection limit down to 4.7 fM, while enhancing the high selectivity, for complementary target DNA with respect to a one-mismatched target. Kafka *et al.* also reported a label-free DNA sensor on a mixed SAM prepared from thiolated probe DNA and 4-mercapto-1-butanol (MCB), which led to the increase in the *R*_ct_ value as well [34]. The mixed system obtained from the solution containing thiolated probe DNA and MCB exhibited the high specificity to the complementary target by showing a large increase in the *R*_ct_ value while a negligible change, or even a decrease, was observed for a single mismatched target.

The SAM prepared using dendrimer molecules is also a useful system for DNA sensing due to their biocompatibility and a sufficient number of functional groups needed for chemical immobilization at the surface. SAMs obtained from the poly(amidoamine) (PAMAM) dendrimer of a spherical shape containing a large number of amine groups at its surface have been characterized and frequently employed for constructing various biosensors by a few groups [35-37]. The Zhu and Fang group immobilized probe DNA using phosphoramidate bonds formed between amine groups of PAMAM and phosphate groups of DNA on the forth-generation PAMAM dendrimer/mercaptoacetic layer on the gold surface [38]. They reported that the target DNA conjugated with the carboxyl-terminated PAMAM dendrimer was hybridized with the probe DNA covalently bound to the mercaptoacetic acid SAM layer on gold [39]. A large increase in *R*_ct_ was detected for complementary hybridization while the change in the charge transfer resistance was so small for hybridization with DNAs containing mismatches. The negatively charged PAMAM dendrimer is helpful for the *R*_ct_ signal amplification due to the enhanced repulsion between the target DNA conjugated with negatively charged PAMAM and the [Fe(CN)_6_]^3−/4−^ redox indicator. As described above, the PAMAM dendrimer can be utilized for the immobilization of probe DNA or the signal enhancement via target-labeling. Probe DNA of an appropriate density obtained by a cone-shaped dendrimer showed very high specificity to the complementary target, leading to different responses of *R*_ct_ values in the presence of [Fe(CN)_6_]^3−/4−^depending on the location of the mismatched pair in the duplex [40]. Thus, various kinds of dendrimers can be diversely utilized for DNA sensors by virtue of their controlled sizes, sufficient functional groups, and unique shapes.

Recently, the Li and Kraatz group took advantage of small molecule [41] and protein [42] binding to ds-DNA to exploit more sensitive detection of single nucleotide mismatches. A small molecule called naphthyridine-azquinolone (Npt-Azq) was found to bind the G-A region of the mismatch, causing a significant change in the *R*_ct_ values for DNA/Npt-Azq films, in which the DNA was a 20-mer containing an A-A mismatch, which allowed the unequivocal detection of the A-A mismatch [41]. The advantage of this approach is that it is specific to the A-A mismatch. Another approach was the use of MutS protein for the detection of mismatches [42]. MutS was found to bind ds-DNA with a single nucleotide mismatch at its top, resulting in a significant increase in Δ*R*_ct_ value. Eight different single-nucleotide mismatches were detected by this method and a C-A mismatch was detectable down to a concentration of as low as 100 pM of the target strand.

## 3. DNA Sensors Based on Conducting Polymers

A conducting polymer containing an extended π-conjugated structure can be very sensitive even to small perturbations at the interface thanks to its high electrical conductivity and electron transfer capabilities when the interface is covered by its film. Biocompatible conducting polymers can be variably modified for immobilization of probe DNA via covalent linking or electrostatic interactions. The conductivity of the conducting polymer electrochemically deposited on the electrode surface can be modulated by changing the pH of the medium, the electrochemical potential, and/or the electrolyte. Because of these characteristics and other advantages, a number of conducting polymers such as polyaniline, polythiophene, polypyrrole, and their derivatives have been utilized extensively for the construction of biosensors including DNA sensors. Rahman *et al.* have recently reviewed biosensors based on conducting polymers [43].

**Figure 3.**
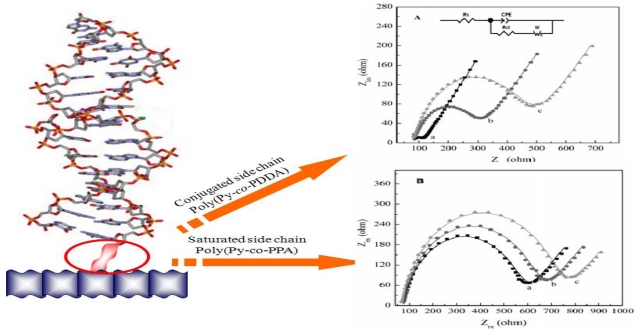
Nyquist plots for electrochemical impedance measurements on electrodes modified with poly(Py-*co*-PPDA) (A) and poly(Py-*co*-PPA) (B) in the presence of 5.0 mM [Fe(CN)_6_]^3−^/[Fe(CN)_6_]^4−^: before (a) and after (b) immobilization of probe DNA, then after incubation (c) with a 20.2 nM DNA target solution [52].

As a simple approach, polypyrrole (PPy) was used directly for the immobilization of a DNA probe based on the electrostatic attraction between electrochemically oxidized-PPy and negatively charged oligonucleotides [44,45]. The enhancement in the *R*_ct_ value was achieved by using CdS nanoparticle tagged-probe DNA for the hybridization of target DNA physically entrapped in the PPy film [46,47]. In these cases, however, the oligonucleotides were randomly attached to the polymer surface showing low hybridization efficiencies. Thus, PPy films derivatized at either the N- or 3-positions were utilized for the construction of DNA sensors [48,49]. Peng *et al.* reported DNA sensors, in which a poly[pyrrole-*co*-4-(3-pyrrolyl) butanoic acid] film, which was electrochemically synthesized from the pyrrole monomer and 4-(3-pyrrolyl) butanoic acid, was used as a DNA immobilization platform [50,51]. On a DNA sensor based on a copolymer film, the *R*_ct_ value was increased with an increase in the target DNA concentration. In agreement with this result, the charge-transfer rate constant to/from the redox couple, [Fe(CN)_6_]^3−/4−^, obtained from Equation 1 using the *R*_ct_ value:

(1) $$k_{a}^{0}=\frac{RT}{n^{2}F^{2}AR_{ct}}\times \frac{1}{C_{0}^{*(1-\alpha )}C_{R}^{*\alpha }}$$

also decreased with an increase in the target DNA concentration. Later, they also reported preparation of variously functionalized PPy copolymers using pyrrole, 3-pyrrolylacrylic acid (PAA), 5-(3-pyrrolyl)-2,4-pentadienoic acid (PPDA), and 3-pyrrolylpentanoic acid (PPA) [52,53]. After the copolymer was electrochemically synthesized at 1.0 V (*vs.* Ag/AgCl), amine-modified probe DNA was covalently attached to the carboxyl group of the side chain of the copolymer through the (1-ethyl-3-(3-dimethylaminopropyl) (EDC) carbodiimide coupling. When the hybridization efficiency was compared with the results obtained by faradaic impedance measurements, the poly(Py-co-PDDA) film containing more conjugated side chains showed the largest increase in *R*_ct_ upon complementary hybridization as shown in Figure 3. This indicates that the polymer backbone conjugated with more probe DNA molecules can be much more susceptible to the change in the DNA probe layer due to the hybridization.

**Scheme 1.**
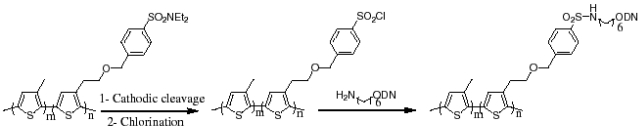
Immobilization of ODN

The detection of the DNA hybridization reaction was also accomplished on the carboxylated polyterthiophene film prepared on the glassy carbon electrode employing non-faradaic impedance spectroscopy without any indicators or labeling molecules present [54]. An amine-modified DNA probe was covalently bound to carboxyl groups on the surface of the functionalized polyterthiophene film. When compared by differences in logarithmic impedances at a fixed frequency of 1.0 kHz, a significant decrease in impedance was observed only for the complementary target, not in the mismatched targets, suggesting that the complementary double-stranded DNA shows an enhanced conductivity in comparison with its single-stranded DNA counterpart. The acrylated polyterthiophene was also used for the DNA sensor through EDC coupling with amine-terminated probe DNA [55,56]. The hybridization efficiency was compared in the reduced and oxidized states of the acrylated polyterthiophene film employing both faradaic and non-faradaic EIS. The result showed that the hybridization was detected more efficiently in the oxidized state of the film displaying a large difference in the phase angle at a fixed frequency. According to Scheme 1, Gautier *et al.* utilized the thiophene matrix covalently coupled with amine modified-probe DNA as a platform for the DNA sensor [57-59]. They reported different responses upon hybridization in terms of the phase angle in Bode plots and the impedance modulus when examined in faradaic and non-faradaic modes. After the hybridization with a complementary target, the impedance modulus was decreased in the non-faradaic mode on the contrary to the increase in the faradaic mode. When the relaxation processes were monitored at 50 Hz and 5 kHz by non-faradaic impedance spectroscopy, the first relaxation process at 50 Hz revealed the hybridization with a decreased phase angle and the second relaxation process at the 5 kHz [57] was related to the length of the target sequence.

Davis *et al.* reported that a highly sensitive 1 fg/mL detection limit was achieved by fabricating the screen-printed electrode modified with electrostatic adsorption of the DNA probes onto the polyethyleneimine film or by incorporating DNA probes into the electrodeposited polyaniline or polydiaminobenzene film [60,61]. They detected the hybridization and discriminated the mismatched DNA against the complementary target on the screen printed electrode modified with various conducting polymers based on the measurement of the mean relative impedance over all frequencies in a non-faradaic mode. To obtain the relative impedance at each frequency, they divided the impedance obtained at a given time interval during the hybridization reaction by the initial impedance value at a selected single frequency. The drop in the impedance observed during the complementary hybridi-zation was caused by the enhanced conductivity of double stranded DNA with respect to that of single strands. Weng *et al.* fabricated the electrochemical DNA sensor on the boron doped diamond (BDD) electrode modified by polyethylimine. The relative impedance at a fixed frequency of 10 Hz without any redox indicator present was decreased with an increase in the target DNA concentration with a good sensitivity with a detection limit of 10^−19^ gm L^−1^ [62]. Gu *et al.* attempted to develop DNA sensors on polyaniline and polyacrylate copolymer modified boron-doped diamond electrodes [63]. On the modified BDD, the detection limit was 2.0 × 10^−8^ M based on the measurement of the decrease in impedances. When the flat-band potential of the BDD was obtained from the Mott-Schottky plot, in which 1/*C*^2^ was plotted against the potential, they observed negative shifts in flat-band potentials upon hybridization with a complementary target, suggesting that the hybridization induce the decreased band bending of the p-type semiconductor. They further reported conducting polymer nanocomposites mixed with nanomaterials such as the carbon-nanotubes and TiO_2_ nanoparticles or nanotubes as a platform for DNA sensors for immobilizing the probe DNA [64-66].

## 4. DNA Sensors Based on Nanomaterials

A variety of nanomaterials such as nanoparticles, nanowires, and nanotubes have been used for constructing DNA sensors. In this section, we briefly review EIS-based DNA sensors constructed using the nanomaterials.

**Scheme 2.**
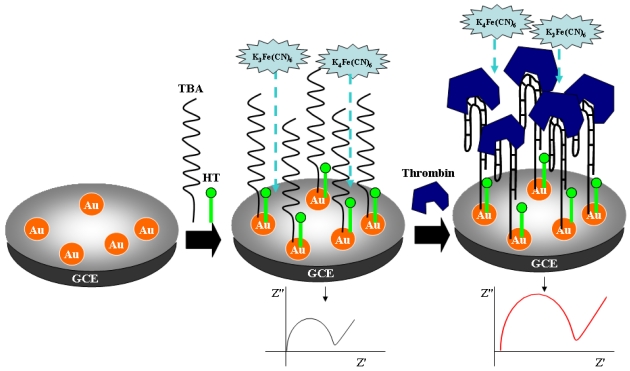
Gold nanoparticles-based aptamer sensor: the thiolated aptamer is first immobilized on the gold nanopartices covering a glassy carbon electrode (GCE), which is then followed by the recognition of thrombin proteins. Here TBA is thrombin aptamer and HT is hexane thiol [75].

Metal nanoparticles have been utilized for enhancing impedance signals by increasing the number of immobilized probe DNA molecules [67]. The Xu and Fang group showed that CdS nanoparticles can be utilized for signal amplification in faradaic-impedance based DNA sensors [68]. A target DNA labeled with CdS nanoparticles was hybridized with amine-modified probe DNA covalently bound to the mercaptoacetic acid monolayer on the gold surface. The *R*_ct_ value for the [Fe(CN)_6_]^3−/4−^ redox indicator was remarkably increased upon hybridization for the complementary target due to the high density negative charges, space blocking, and semiconducting properties of the CdS nanoparticles, which lowered the detection limit down to 1.43 × 10^−10^ M. Also, gold nanoparticles deposited on the gold electrode were employed as a platform for the immobilization of thiolated probe DNA [69]. The difference in *R*_ct_ values before and after hybridization showed a linear relation with the concentration of the target in a range of 2.0 × 10^−12^ ∼ 9.0 × 10^−8^ M. Bonnani *et al.* utilized a streptavidin-coated gold nanoparticles for improving the *R*_ct_ signal [70]. The probe DNA immobilized on the graphite epoxy composite working electrode by simple physical adsorption was hybridized with a biotinylated complementary oligomer, followed by binding to streptavidin-coated gold nanoparticles for an improvement of the *R*_ct_ signal. The relative *R*_ct_ variance for the [Fe(CN)_6_]^3−/4−^ pair was magnified with an increase in the target concentration due to the electrostatic repulsion and steric hindrances. The detection limit was improved from 26.5 pmol to 11.8 pmol in comparison to the non-amplification method. In their more recent report, they described the immobilization of the amine-terminated probe DNA by carbodiimide chemistry on the screen-printed electrode, which was modified by carboxylated multi-walled carbon nanotubes [71]. Based on the same amplification technique of streptavidin-coated gold nanoparticles, they obtained a magnificently enhanced detection limit of 22 fmol on the multi-wall carbon nanotube-modified screen printed electrode. In addition to this, the signal enhancement technique using gold nanoparticles have been frequently applied to aptamer sensors recognizing the specific proteins as well as DNA [72-75]. The Li and Zhang group utilized gold nanoparticles electrodeposited on the glassy carbon electrode for the immobilization of the thiolated aptamer and the signal enhancement according to Scheme 2 [75]. Increases in *R*_ct_ values were monitored in a concentration range of thrombin from 0.12 nM to 30 nM. Using the [Fe(CN)_6_]^3−/4−^ redox indicator, Deng *et al.* detected thrombin through the increase in *R*_ct_ on the sandwich system of aptamer/thrombin/ aptamer functionalized Au nanoparticles for a cascaded signal amplification [76]. The three level amplification was accomplished by the attachment of aptamer-functionalized Au nanoparticles as a first step, the steric hindrance by the enlargement of aptamer Au nanoparticles as a second step, and the increased electrostatic repulsion by the sodium dodecylsulfate (SDS) stabilizer of aptamer-Au nanoparticles as a final step; this series of procedures enabled a detection limit of 100 fM to be obtained. Magnetic nanoparticles were also used as a DNA binding platform as gold nanoparticles.

Biotin-modified hepatitis-B virus (HBV) probe DNA was immobilized on the streptavidin coated magnetic nanoparticles by the streptavidin-biotin interaction. The magnetic nanoparticles modified with HBV probe DNA was attached to the gold surface, and the hybridization with the target HBV sequence was detected by an increase in resistance using non-faradaic EIS, which led to the detection limit of 50 pmol for a 20 μL sample [77]. Another example of the nanoparticle platform as a sensing material is praseodymium oxide nanoparticles with a high dielectric constant, a large band gap, and a high electron affinity [78]. Thiol modified probe DNA attached to the amine-modified praseodymium oxide nanoparticles was allowed to hybridize with its target DNA, which was detected by the decrease in capacitance without a redox indicator present.

**Scheme 3.**
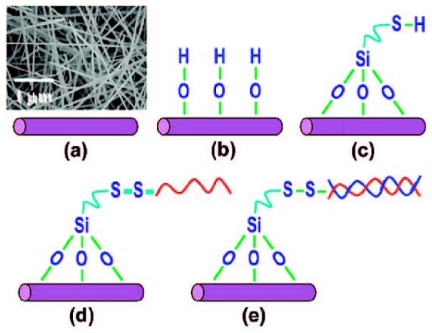
Schematic diagram of a DNA sensor based on GaN nanowires: (a) nanowires, (b) hydroxylation of the surfaces, (c) modification by 3-mercaptopropyl trimethoxysilane (MPTS), and (d) immobilization of probe DNA, and e) hybridization [82].

**Scheme 4.**
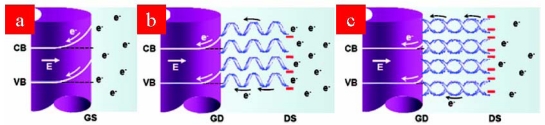
The band-bending of GaN nanowires: (a) bare GaN nanowires in contact with the electrolyte, (b) reduced band-bending due to the immobilization of probe DNA, and (c) further flattening of the band-bending by hybridization. Here GS represents the GaN/solution interface, GD the GaN/DNA interface, and DS the DNA/electrolyte interface [82].

Chemically stable and biocompatible GaN nanowires have been widely investigated for various applications to biotechnology [79-81]. GaN nanowires have high conductivity due to the substantial charge separation between the surface and the core; thus, strongly negatively charged DNA can induce large changes in the electrochemical behavior of the nanowires. Chen *et al.* developed a novel DNA sensor based on GaN nanowires as shown in Scheme 3 [82]. The electrical properties of nanowires thus modified were studied by non-faradaic impedance spectroscopy before and after the DNA hybridization. As shown in Scheme 4, the upward band-bending of the as grown GaN nanowires was flattened by complementary hybridization resulting in a decrease in the resistance or the barrier at the GaN/DNA interface. The resistance was shown to be further decreased with an increase in the target DNA concentration down to pico to micromolar levels.

Finally, carbon nanotubes (CNTs) are also one of important candidates for constructing DNA sensors [83-86]. Various applications of CNTs and the related methodologies have been reviewed [86]. Jiang *et al.* first electropolymerized L-lysine to prepare its polymeric film on carboxylated carbon nanotubes spread on a carbon paste electrode (CPE). The probe DNA was then immobilized on the poly-L-lysine/CNT/CPE by electrostatic attraction. The hybridization of the PAT (phosphinothricin acetyltransferase) gene fragment was monitored by an increase in *R*_ct_ values by the faradaic EIS using the [Fe(CN)_6_]^3−/4−^ redox indicator [87] (detection limit to 3.1 × 10^−13^ mol/L). In addition, a nanocomposite membrane consisting of nanoshuttle-shaped cerium oxide (CeO_2_) particles, single-walled carbon nanotubes and 1-butyl-3-methylimidazolium hexafluorophosphate was also investigated on a glassy carbon electrode for DNA immobilization and its hybridization using the faradaic EIS technique. This nanocomposite system remarkably improved the sensitivity of DNA hybridization detection based on the increase of *R*_ct_ for the [Fe(CN)_6_]^3−/4−^ indicator after hybridization showing a detection limit of 2.3 × 10^−13^ M [88].

## 5. Amperometric and Voltammetric Detection Versus the EIS Detection

**Figure 4.**
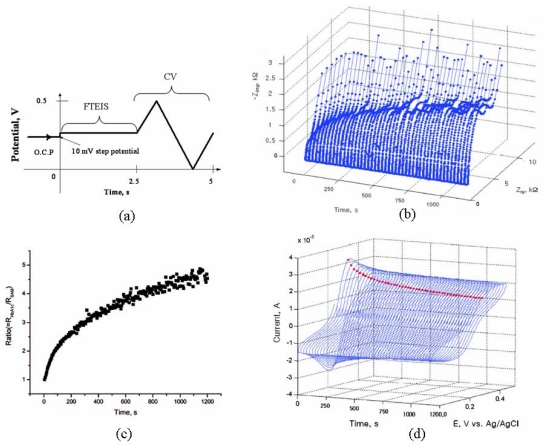
Comparison of impedance and CV measurements during the reaction of HbA_1c_ with the thiophene-3-boronic acid SAM [90]: (a) the waveform applied to the electrode to sequentially obtain FTEIS and CV data, (b) a series of Nyquist plots, (c) the ratio of *R*_ct(t)_ and *R*_ct,initial_, and (d) a series of CVs. Note the 480% increase in impedance *vs.* the 46% decrease in peak heights.

While we have been describing only the EIS method of detection for the DNA sensors thus far, both amperometric and voltammetric detection methods had widely been used in earlier studies [89]. Although no direct comparison of these detection methods with the EIS technique is available for DNA sensors, recent studies on other biosensors demonstrate that the EIS detection is much preferred to other electrochemical methods including amperometric, voltammetric, and/or potentiometric methods. In recent studies, Park *et al.* demonstrated that EIS measurements can provide significantly more sensitive responses than cyclic voltammograms (CVs) do [90,91]. In one example, they monitored the kinetics for the esterification reaction between boronic acid, which is immobilized on the gold electrode via the formation of a thiophene-3-boronic acid SAM, and glycated hemoglobin (HbA_1c_) in solution with the [Fe(CN)_6_]^3−/4−^ indicator present to probe how far the reaction proceeded [90]. When the HbA_1c_ molecule reacts with boronic acid on the electrode, its surface would be effectively blocked. In their experiments, both impedance and cyclic voltammetric measurements were made in real time sequentially by applying the waveform shown in Figure 4a, in which a 10 mV step was applied for the first 2.5 s to record an impedance spectrum in a full frequency region from about 10 kHz down to 0.40 Hz, followed by a ramp signal for the next 2.5 s to record CVs. The Fourier transform EIS (FTEIS), in which a full EIS spectrum is acquired from a small potential step signal by going through a number of procedures, have been well established via a series of publications [15,92-96]. Figures 4b,c show a series of impedance spectra and CVs thus obtained, respectively, both at every 2.5 s. While CVs show a maximum decrease in their peak heights by only about 46% in 20 min, the *R*_ct_ value showed a maximum increase by about 480% during the same period while the reaction was proceeding after a solution containing HbA_1c_ was added to the boronic acid covered electrode immersed in the solution. A very similar observation was reported for a serotonin sensor, in which an (*R*)-lipo-diaza-18-crown-6 SAM was used to capture serotonin molecules in the presence of the [Fe(CN)_6_]^3−/4−^ redox indicator [91]. In the latter case, however, the *R*_ct_ value was decreased upon addition of serotonin instead due to a different chemistry taking place from that for the HbA_1c_ sensor. Both cases indicate that monitoring the change in *R*_ct_ values is much more sensitive than monitoring currents by voltammetric or amperometric methods. We should also point out that the FTEIS technique, although we have not described in detail here, allows one to record a full spectrum in a short period, as short as less than 0.1 s depending on the electrochemical reversibility of the redox indicator used [92-96].

## 6. Conclusions

We have briefly reviewed recent advances made in DNA sensors employing EIS as a detection tool among many other biosensors using similar platforms [97-101]. Three main types of platforms such as SAMs, conducting polymers, and metal or semiconductor nanoparticles have been covered in this review. As pointed out, the selectivity is provided by choosing an appropriate chemistry taking place on the electrode surface, which renders the EIS measurements label free. In this area, working out a creative chemistry on the electrode surface is a key strategy to developing more selective and sensitive DNA or other biosensors. We have also demonstrated that the EIS method can be significantly more sensitive than their traditional counterparts such as amperometric, voltammetric, and potentiometric measurements as a detection tool.

Despite the advantages of EIS as a detection tool described earlier in this review, it also suffers from a few disadvantages and limitations. In most cases, its sensitivity still cannot match that of the fluorescence detection as the sensitivity of the latter method is directly related to the intensity of the excitation source. Another limitation arises from the requirement that the EIS detection must be used only at an electrode, while any surfaces may serve as a platform for the fluorescence detection. Again, we described how the surface chemistry can enhance the sensitivity as well as the selectivity.

It often takes too long to acquire an EIS spectrum in a reasonably wide frequency range employing the traditional frequency response analyzer. For this reason, a series of relative values of impedances or capacitances at a fixed frequency have been frequently utilized for real-time sensing [54,57], which is useful for improving the binding/unbinding accuracy and determining the affinity constant. However, we also notice that FTEIS experiments reduce the impedance measurement times by orders of magnitudes [88-92]. It is thus possible to use the EIS measurements for real-time monitoring of the reaction kinetics at the electrode surface and also as a detection tool when scanning an array of hundreds of electrodes on a DNA chip. The next step of the development of the DNA or other biochips should be the incorporation of the capability of making real time measurements so that many DNA or other samples would be analyzed by simply scanning across the chip. More work on the use of nanostructures to enhance the sensitivity of biosensors, as well as the incorporation of FTEIS for analyzing a large number of samples, should represent an important breakthrough to be achieved in the not too distant time to come.
